# Comparative Efficacy and Safety of PARP Inhibitors as Maintenance Therapy in Platinum Sensitive Recurrent Ovarian Cancer: A Network Meta-Analysis

**DOI:** 10.3389/fonc.2020.573801

**Published:** 2021-02-22

**Authors:** Yangchun Xu, Lei Ding, Yuan Tian, Miaomiao Bi, Ning Han, Ling Wang

**Affiliations:** ^1^ Department of Dermatology, The Second Hospital of Jilin University, Changchun, China; ^2^ Department of Radiology, China-Japan Union Hospital of Jilin University, Changchun, China; ^3^ Department of Medical Examination, China-Japan Union Hospital of Jilin University, Changchun, China; ^4^ Department of Ophthalmology, China-Japan Union Hospital of Jilin University, Changchun, China; ^5^ Department of Gynecology and Obstetrics, The Second Hospital of Jilin University, Changchun, China

**Keywords:** PARP inhibitor, platinum, ovarian cancer, network meta-analysis, progress-free survival

## Abstract

This meta-analysis investigated the comparative efficacy and safety of PARP inhibitor monotherapy as maintenance treatment in platinum sensitive recurrent ovarian cancer (ROC). Electronic databases were systematically searched for relevant RCTs. The primary endpoint was PFS. The results were stratified based on three categories: BRCA mutated patients, HRD patients, and overall population. The secondary outcome were discontinuations due to adverse events and grade 3 or 4 adverse events in maintenance phase. Five eligible RCTs were included in the network meta-analysis. For patients with BRCA mutated ovarian cancer, olaparib-throughout (HR = 0.21 with 95% CrI: 0.081–0.55), rucaparib (HR = 0.23 with 95% CrI: 0.16–0.34), olaparib (HR = 0.27 with 95% CrI: 0.20–0.35), and niraparib (HR = 0.26 with 95% CrI: 0.17–0.41) were all highly effective in comparison with placebo at improving PFS. For HRD patients, both rucaparib (HR = 0.32 with 95% CrI: 0.24–0.42) and niraparib (HR = 0.38 with 95% CrI: 0.24–0.60) were all highly effective in comparison with placebo at improving PFS. For the overall population, olaparib-throughout (HR = 0.51 with 95% CrI: 0.34–0.76), rucaparib (HR = 0.37 with 95% CrI: 0.30–0.45), olaparib (HR = 0.35 with 95% CrI: 0.25–0.49), and niraparib (HR = 0.38 with 95% CrI: 0.30–0.48) were all highly effective in comparison with placebo at improving PFS. Regarding grade 3 or 4 adverse events, the incidence of grade 3 or 4 toxicity reactions to rucaparib and niraparib were significantly higher than in the olaparib group. In terms of discontinuations due to adverse events, the treatment discontinuations were not significantly different between the three drugs. In summary, all the included maintenance treatment regimens are effective regardless of BRCA mutational status, and no statistically significant differences between rucaparib, niraparib and Olaparib in terms of PFS. In terms of safety profile, the three drugs present manageable adverse events. Clinicians should consider potential adverse events related to each of these interventions in clinical practice, and the adverse events are generally manageable.

## Introduction

Ovarian cancer is the eleventh most common cancer worldwide and the fifth leading cause of cancer-related death (1). Although most patients with advanced ovarian cancer respond to initial platinum-based chemotherapy following cytoreductive surgery, approximately 70% will experience relapse and require subsequent therapies. ROC cannot be cured, with most patients receiving multiple treatment lines before ultimately dying from the disease (2). Given the deeply researching of molecular pathways found to be dysregulated during the multistep process of oncogenesis, many therapeutic targets have been identified and gave significant results in the clinical practice, which driven the management of cancer into individualized treatments. Poly(ADP-ribose) polymerase inhibitors are one of new personalized treatments for patients with high-grade serous ovarian cancer and demonstrate a high survival advantage in several randomized controlled trials (RCTs) and meta-analyses (3–6). The treatment modality is based on the mechanisms of “synthetic lethal” and “PARP trapping”, especially for patients with homologous recombination deficiencies (HRD) (7).

PARP inhibitors currently used for maintenance treatment for platinum sensitive ROC include olaparib, rucaparib, and niraparib. The three drugs had been approved from December 2014 to July 2017 for the treatment of ROC (5) and recommended as maintenance therapy for platinum sensitive ROC by the NCCN guideline (8). However, all PARP inhibitors have never been compared with each other because of the lack of head-to-head trials. Although recent traditional meta-analyses have been published on PARP inhibitors as maintenance treatment in platinum sensitive ROC (3–6), comparisons among the three drugs were little explored because of the limitation of traditional meta-analysis methods which is based on direct evidence (9). Thus, the comparative efficacy and safety of FDA-approved PARP inhibitors as maintenance treatment in platinum sensitive ROC is still unknown.

To provide concrete evidence for clinical practice, there is an urgent need for a thorough comparison of survival and safety profile. Herein, we performed a network meta‐analysis to compare the effectiveness and safety of FDA-approved PARP inhibitors (olaparib, rucaparib, and niraparib) as maintenance therapy in platinum sensitive ROC.

## Materials and Methods

This study followed Preferred Reporting Items for Systematic Reviews and Meta-analyses (PRISMA) extension for Network Meta-Analysis (10).

### Literature Search

A literature search was conducted on PubMed, Embase and the Cochrane Central Register in May 2020. The reference lists of studies identified through the initial screening were used to identify trials missed by the computerized database search. The following search terms were used: olaparib, niraparib, and rucaparib, PARP inhibitors, maintenance therapy, recurrent, and ovarian cancer.

### Eligibility and Exclusion Criteria

The inclusion criteria were as follows. Participants: Patients with platinum sensitive ROC. Intervention: A history of FDA-approved PARP inhibitor (such as olaparib, niraparib, and rucaparib) as maintenance therapy for ROC. Comparators: placebo or another PARP inhibitor. Outcome: The primary outcome was progression free survival (PFS), defined as the time between randomization and either disease progression or death; The safety outcomes were discontinuations due to adverse events and the grade 3 or 4 adverse events in maintenance phase which were assessed with The Common Terminology Criteria for Adverse Events (CTCAE). CTCAE is the most used tool for evaluating adverse event type and severity in clinical practice with a grading scale and clear definitions. Grade 3 and grade 4 adverse events indicate severe and life-threatening toxicity, respectively (11). Study design: published randomized controlled trials (RCTs).

The exclusion criteria were as follows: (1) any non-approved PARP inhibitor; (2) patients without platinum sensitive ROC; (3) non‐RCTs, reviews or reports solely focusing on laboratory findings; (4) meeting abstracts; (5) animal‐only experiments; and (6) studies reported in a language other than English.

### Study Selection and Data Extraction

Two reviewers independently screened the titles and abstracts of the literature identified with the search strategy. Multiple publications of the same RCT were counted only once to avoid double‐counting of patients. A predefined data extraction sheet, which was designed according to the Cochrane Handbook (12), was used to extract characteristics and outcomes of selected RCTs, including author information, study design, participant characteristics, interventions, number of patients by BRCA mutational status, discontinuation due adverse events, frequency of grade 3 or 4 adverse events, and other information as needed. Authors were directly contacted to seek additional information in cases where the data were unclear or not reported. Any discrepancies were resolved by consensus and arbitration by the whole review team.

### Risk of Bias Assessment

Two independent reviewers evaluated the methodological quality all included trials according to the Cochrane Handbook (12). Risk of bias were assessed based on the selection bias, performance bias, detection bias, attrition bias, and reporting bias. Disagreements were resolved by discussion and consensus among all authors.

### Statistical Analysis

We used fixed effects network meta-analysis to combine direct and indirect evidence of all treatment effects using R software. Then, we calculated hazards ratios (HRs) with their 95% credible intervals (CrIs) for the survival outcome. The efficacy analyses were done on the intention-to-treat population; Subgroup analyses were performed based on the following groups: BRCA mutated patients, HRD patients, and the overall population. Safety analyses included patients who received at least one dose of study treatment. We calculated risk ratios (RRs) with their 95% CrIs for the safety outcomes.

To rank the intervention, we calculated the probabilities of the surface under the cumulative ranking curve (SUCRA) (13).

## Results

### Characteristics of Included Studies

**Figure 1:**
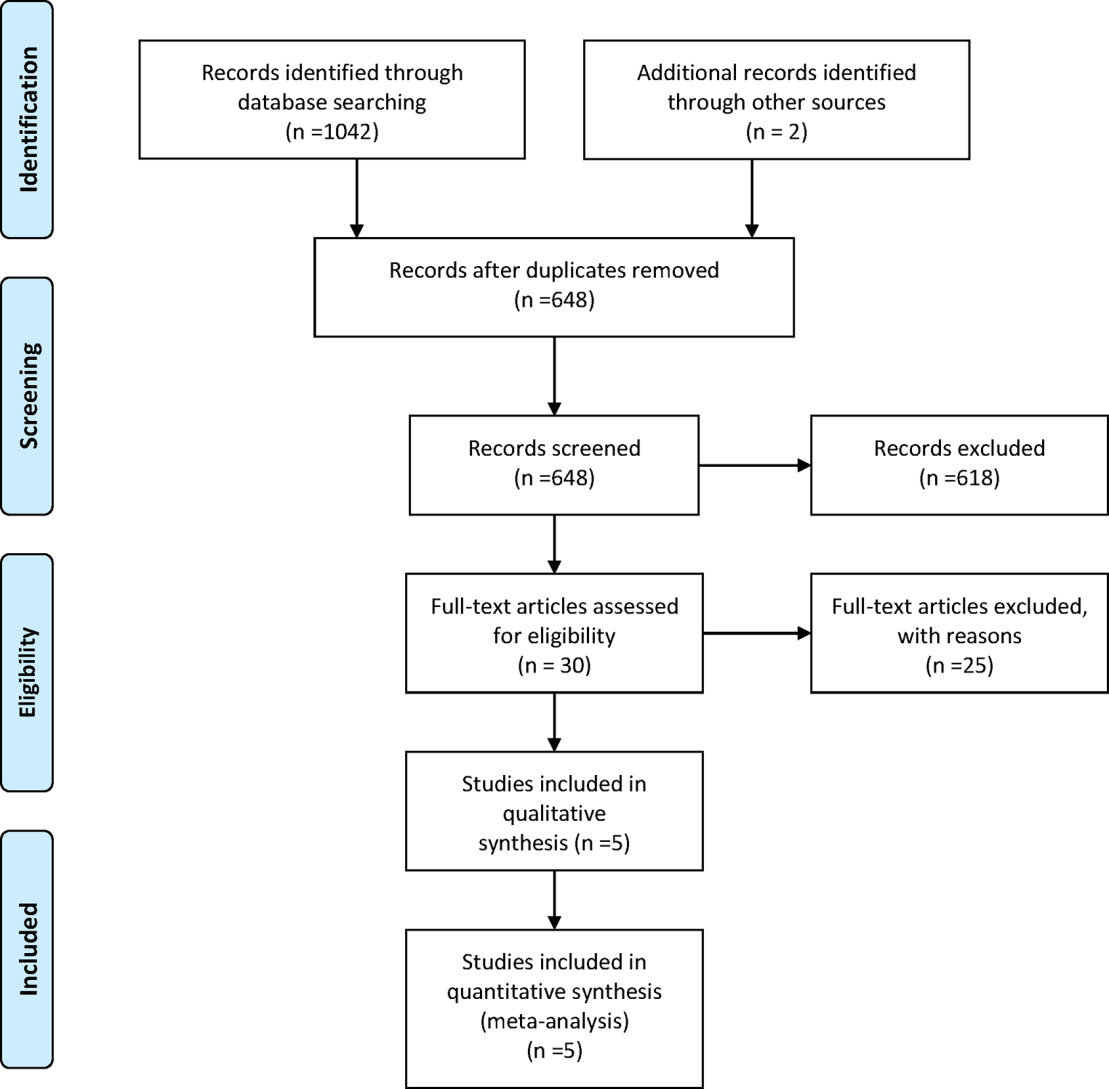
Study Flow Diagram.

**Figure 2:**
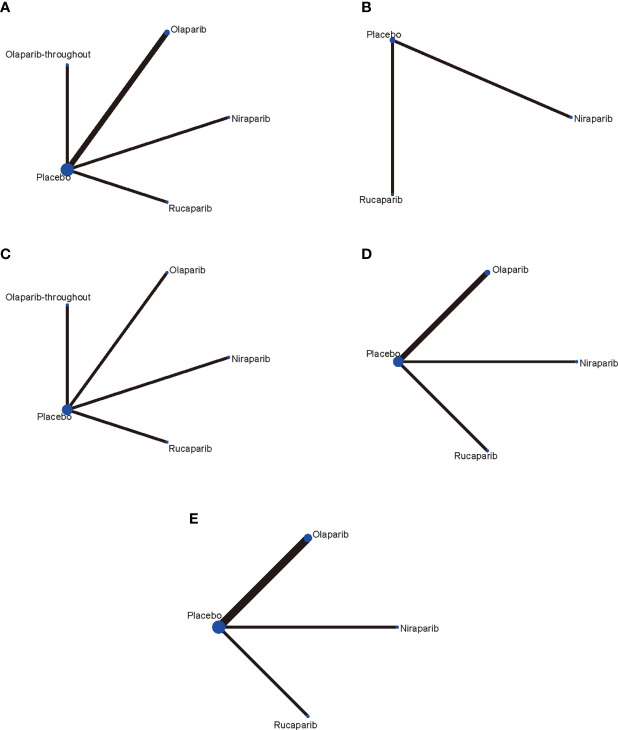
Network of the comparisons for the network meta-analysis. **(A)** PFS in BRCA mutated patients; **(B)** PFS in HRD positive patients; **(C)** PFS in overall population; **(D)** Network meta‐analysis of discontinuations due to adverse events; **(E)** Network meta‐analysis of adverse events in maintenance phase. Each circular node represents a type of treatment. The circle size is proportional to the total number of studies (under the drug name). The width of lines is proportional to the number of studies performing head-to-head comparisons in the same study.

Five RCTs (14–18) involving 1,838 patients were included in the current study. It should be noted the trial NCT01081951 investigated the efficacy and tolerability of olaparib plus chemotherapy, followed by olaparib monotherapy as a maintenance treatment (15). Thus, we presented the treatment in NCT01081951 as “olaparib-throughout”. The flowchart of the literature search and selection process is shown in **Figure 1**. The features of the included trials are summarized in **Table 1**. Although studies comparing different agents were included, the baseline characteristics were similar among all the trials. Most of the studies included both mutated and not mutated patients; only the trial reported by Pujade-Lauraine E et al. included exclusively BRCA mutated population (16). A graphical network structure shows the network of trials for different primary and secondary outcomes (**Figure 2**). Each circular node represents a type of treatment. The circle size is proportional to the total number of studies (under the drug name). The width of lines is proportional to the number of studies performing head-to-head comparisons in the same study (19).

**Table 1:** Characteristics of the trials included in the meta-analysis.

Author(Trial)	Year	Phase	Agents in maintenance phase	Number of BRCAm Pts in efficacy analyses *	Number of HDR positive Pts in efficacy analyses*	Number of overall Pts in efficacy analyses*	Mean age(years)	Number of overall Pts in safety analyses^†^	Discontinuations due to adverse events- no (%)	Grade 3 or 4 adverse events in maintenance phase- no (%)	Treatment-related death	Outcomes(Subgroup)
**Friedlander et al. (** 14 **)** **(STUDY 19)**	2018	II	Olaparib	74	NR	136	58	136	8 (5.8)	59 (43)	2	•PFS (BRCAm/whole population) •Adverse Events •Discontinuations due to adverse events
			Placebo	62	NR	128	59	128	2 (1.5)	28 (22)	0	
**Pujade-Lauraine et al. (** 16 **)** **(SOLO2)**	2017	III	Olaparib	196	NA	196	56	195	21 (10.8)	71 (36)	1	•PFS (BRCAm) •Adverse Events •Discontinuations due to adverse events
			Placebo	99	NA	99	56	99	2 (2.0)	18 (18)	0	
**Coleman et al. (** 17 **)** **(ARIEL3)**	2017	III	Rucaparib	130	236	375	61	372	50 (13.4)	209 (56)	2	•PFS (BRCAm/HRDp/whole population) •Adverse Events •Discontinuations due to adverse events
			Placebo	66	118	189	62	189	3 (1.6)	28 (15)	0	
**Mirza et al. (** 18 **)** **(ENGOT-OV16/NOVA)**	2016	III	Niraparib	138	244	372	NR	367	54 (14.7)	272 (74)	1	•PFS (BRCAm/HRDp/whole population) •Adverse Events •Discontinuations due to adverse events
			Placebo	65	121	181	NR	179	4 (2.2)	41 (23)	1	
**Oza et al. (** 15 **)**	2015	II	Olaparib	20	NR	81	59	66	5 (7.6)	19 (29)	0	•PFS (BRCAm/whole population) •Adverse Events
			No treatment	21	NR	81	62	55	NA	9 (16)	0	

Pts, patients; BRCAm, BRCA mutated; HRDp, homologous recombination deficiency positive; PFS, progression-free survival; OS, overall survival.

*The efficacy analyses were done on the intention-to-treat population.

^†^The safety analyses included patients who received at least one dose of study treatment.

The “Risk of bias table” (**Supplement Table 1**) illustrated the risk of bias, which was globally low.

### Network Meta‐Analysis of PFS in BRCA Mutated Patients

**Figure 3:**
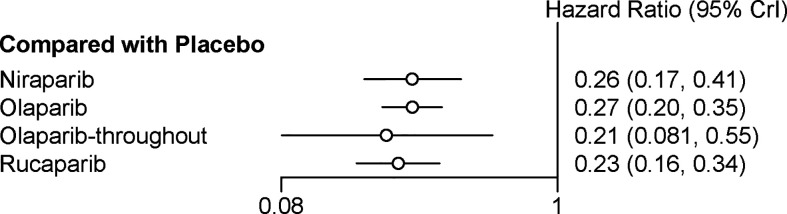
Network meta‐analysis of PFS in BRCA mutated patients.

All the five trials contributed to our network meta-analysis of PFS in BRCA mutated patients, comparing the five treatments. For patients with BRCA mutated ovarian cancer, chemotherapy plus olaparib followed by olaparib maintenance (Olaparib-throughout) (HR = 0.21 with 95% CrI: 0.081–0.55), rucaparib (HR = 0.23 with 95% CrI: 0.16–0.34), olaparib (HR = 0.27 with 95% CrI: 0.20–0.35), and niraparib (HR = 0.26 with 95% CrI: 0.17–0.41) were all highly effective in comparison with placebo at improving PFS (**Figure 3** and **Table 2**). No treatment was clearly superior to others between olaparib-throughout, olaparib, rucaparib, and niraparib. According to the SUCRAs, the rank probability of PFS in BRCA mutated patients was as follows: olaparib-throughout (73.1%) > rucaparib (69.7%) > niraparib (54.4%) > olaparib (52.8%) > placebo (0.013%).

**Table 2:** Network meta‐analysis of PFS in BRCA mutated patients.

**Niraparib**				
1.0 (0.59, 1.7)	**Olaparib**			
1.3 (0.44, 3.6)	1.3 (0.46, 3.4)	**Olaparib-throughout**		
1.1 (0.64, 2.0)	1.1 (0.71, 1.8)	0.90 (0.32, 2.5)	**Rucaparib**	
**0.26 (0.17, 0.41)**	**0.27 (0.20, 0.35)**	**0.21 (0.081, 0.55)**	**0.23 (0.16, 0.34)**	**Placebo**

Results in each cell represent the pooled HR and its 95% CrI. The estimates are located at the crossing between the column‐defining treatment and row‐defining treatment. An HR lower than one favors the column‐defining treatment. The significant results are presented in bold.

### Network Meta‐Analysis of PFS in HRD Positive Patients

**Figure 4:**
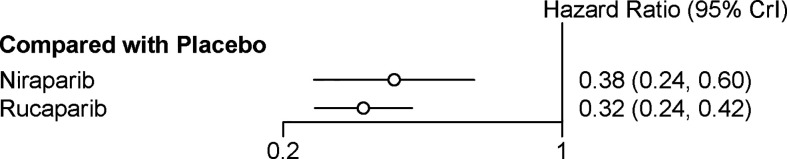
Network meta‐analysis of PFS in HRD positive patients.

Two trials contributed to our network meta-analysis of PFS in HRD positive patients, comparing the three treatments (rucaparib, niraparib, and placebo) (17, 18). For HRD patients, both rucaparib (HR = 0.32 with 95% CrI: 0.24–0.42) and niraparib (HR = 0.38 with 95% CrI: 0.24–0.60) were all highly effective in comparison with placebo at improving PFS (**Figure 4** and **Table 3**). According to the SUCRAs, the rank probability of PFS in HRD positive patients was as follows: rucaparib (87.0%) > niraparib (63.0%) > placebo (0.003%).

**Table 3:** Network meta‐analysis of PFS in HRD positive patients.

**Niraparib**		
1.2 (0.70, 2.0)	**Rucaparib**	
**0.38 (0.24, 0.60)**	**0.32 (0.24, 0.42)**	**Placebo**

Results in each cell represent the pooled HR and its 95% CrI. The estimates are located at the crossing between the column‐defining treatment and row‐defining treatment. An HR lower than one favors the column‐defining treatment. The significant results are presented in bold.

### Network Meta‐Analysis of PFS in Overall Population

**Figure 5:**
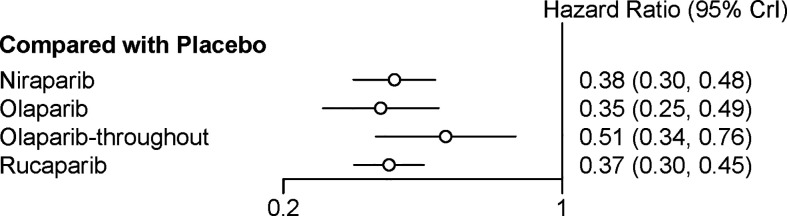
Network meta‐analysis of PFS in overall population.

Four trials contributed to our network meta-analysis of PFS in whole population, comparing all the five treatments (14, 15, 17, 18). For the whole population, olaparib-throughout (HR = 0.51 with 95% CrI: 0.34–0.76), rucaparib (HR = 0.37 with 95% CrI: 0.30–0.45), olaparib (HR = 0.35 with 95% CrI: 0.25–0.49), and niraparib (HR = 0.38 with 95% CrI: 0.30–0.48) were all highly effective in comparison with placebo at improving PFS (**Figure 5** and **Table 4**). No treatment was clearly superior to others between olaparib-throughout, olaparib, rucaparib, and niraparib. According to the SUCRAs, the rank probability of PFS in whole population was as follows: olaparib (79.1%) > rucaparib (72.5%) > niraparib (66.7%) > olaparib-throughout (31.6%) > Placebo (0.016%).

**Table 4:** Network meta‐analysis of PFS in overall population.

**Niraparib**				
1.1 (0.72, 1.6)	**Olaparib**			
0.74 (0.47, 1.2)	0.69 (0.41, 1.2)	**Olaparib-throughout**		
1.0 (0.76, 1.4)	0.95 (0.64, 1.4)	1.4 (0.88, 2.2)	**Rucaparib**	
**0.38 (0.30, 0.48)**	**0.35 (0.25, 0.49)**	**0.51 (0.34, 0.76)**	**0.37 (0.30, 0.45)**	**Placebo**

Results in each cell represent the pooled HR and its 95% CrI. The estimates are located at the crossing between the column‐defining treatment and row‐defining treatment. An HR lower than one favors the column‐defining treatment. The significant results are presented in bold.

### Network Meta‐Analysis of Discontinuations Due to Adverse Events

**Figure 6:**
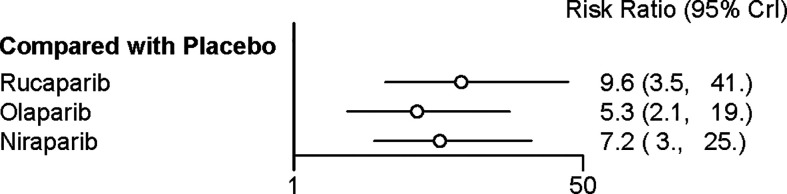
Network meta‐analysis of discontinuations due to adverse events.

Four trials contributed to our network meta-analysis of adverse events in maintenance phase, comparing the four treatments (16–18, 20). Regarding discontinuations due to adverse events in maintenance phase, compared with placebo, rucaparib (RR = 9.6 with 95% CrI: 3.5–41), niraparib (RR = 7.2 with 95% CrI: 3–25), and olaparib (RR = 5.3 with 95% CrI: 2.1–19) led to a significantly higher risk of discontinuations due to adverse events (**Figure 6** and **Table 5**). No significant differences in discontinuation rate were found between olaparib, rucaparib, and niraparib. According to the SUCRAs, the rank probability of discontinuations due to adverse events was as follows: rucaparib (80.3%) > niraparib (67.5%) > olaparib (52.1%) > placebo (0).

**Table 5:** Network meta‐analysis of discontinuations due to adverse events (above the diagonal) and adverse events in maintenance phases (below the diagonal).

**Niraparib**	1.4 (0.30, 6.3)	0.75 (0.14, 3.6)	**7.2 (3, 25)**
**1.6 (1.1, 2.4)**	**Olaparib**	0.55 (0.10, 2.8)	**5.3 (2.1, 19)**
0.85 (0.53, 1.3)	**0.52 (0.33, 0.80)**	**Rucaparib**	**9.6 (3.5, 41)**
**3.3 (2.5, 4.4)**	**2 (1.5, 2.6)**	**3.8 (2.8, 5.6)**	**Placebo**

Results in each cell represent the pooled RR and its 95% CrI. The estimates are located at the crossing between the column‐defining treatment and row‐defining treatment. For discontinuations due to adverse events, an RR lower than one favors the row‐defining treatment. For adverse events in maintenance phases, an RR lower than one favors the column‐defining treatment. The significant results are presented in bold.

### Network Meta‐Analysis of Adverse Events in Maintenance Phase

**Figure 7:**
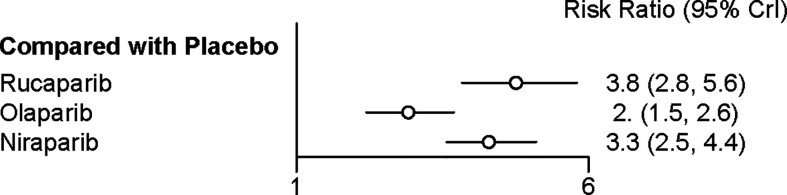
Network meta‐analysis of adverse events in maintenance phase.

All the five trials contributed to our network meta-analysis of adverse events in maintenance phase, comparing the five treatments. Regarding grade 3 or 4 adverse events in maintenance phase, compared with placebo, rucaparib (RR = 3.8 with 95% CrI: 2.8–5.6), olaparib (RR = 2 with 95% CrI: 1.5–2.6), and niraparib (RR = 3.3 with 95% CrI: 2.5–4.4) led to a numerically higher risk of grade 3 or 4 adverse events (**Figure 7** and **Table 5**). Besides, both rucaparib (RR = 1.9 with 95% CrI: 1.3–3.1) and niraparib (RR = 1.6 with 95% CrI: 1.1–2.4) showed a higher risk of grade 3 or 4 adverse events as compared with olaparib. According to the SUCRAs, the rank probability of grade 3 or 4 adverse events in maintenance phase was as follows: placebo (100.0%) > olaparib (66.4%) > niraparib (25.7%) > rucaparib (7.8%).

## Discussion

Based on the concept of synthetic lethality, PARP inhibitors were developed to treat patients with HRD, specifically for BRCAm patients. Evidence from clinical trials and meta-analyses to date has shown that BRCAm patients derive the greatest benefit from PARP inhibitors as maintenance therapy. However, the comparative efficacy and safety of individual FDA-approved PARP inhibitors as maintenance treatment in platinum sensitive ROC is still unknown.

Similar to previous meta-analyses (5, 6), the results of the current work confirm the efficacy of all the included PARP inhibitors in improving PFS when administered to platinum sensitive ROC patients regardless of BRCA mutational status in a maintenance setting. The results partially support the concept of synthetic lethality.

Furthermore, we explored the use of different PARP inhibitors as monotherapy, comparing them among each other in terms of efficacy, discontinuations due to adverse events, and toxicity. We found no statistically significant differences between rucaparib, niraparib and olaparib in terms of PFS. Although olaparib-throughout treatment could significantly improve PFS, olaparib-throughout treatment was not superior to olaparib maintenance treatment alone, suggesting that the maintenance phase was probably the key contributor to the improvement in PFS. Oza, A. M. et al. held that the late separation of the PFS curves and improvement in objective response during the combination phase might suggest that the benefit from olaparib is related to its use as maintenance therapy (15). Based on these findings, the combination of olaparib plus chemotherapy is not believed to provide an advantage over olaparib maintenance therapy alone.

Regarding grade 3 or 4 adverse events in maintenance phase, compared with placebo, all the investigated PARP inhibitors were associated with a higher rate of adverse events in maintenance phase. In addition, the incidence of grade 3 or 4 toxicity reactions to rucaparib and niraparib were significantly higher than in the olaparib group. In terms of discontinuations due to adverse events, the findings suggest that all the three PARP inhibitors led to higher discontinuation rates due to adverse events than placebo. The treatment discontinuations were not significantly different between the three drugs, although the fewest grade 3 or 4 adverse events was associated with olaparib. Thus, even though the odds of experiencing grade 3 or 4 adverse events were not comparable to olaparib, adverse events in rucaparib and niraparib were generally managed. Management of adverse events included supportive care and dose modifications (including treatment interruption or dose reduction) (17, 21).

Notably, although higher grade 3 or 4 adverse events and discontinuation rates are associated with the three drugs compared to placebo, the trials concerning olaparib and niraparib found no difference in quality-of-life indicators over time as patients in placebo group have earlier progression and symptoms related to cancer (14, 16, 18).

The present network meta-analysis should be interpreted with caution in view of the following limitations. Firstly, this network meta-analysis was conducted at the study level and may not reflect the confounding variables that would be present at the patient level. For example, none of the included trials report the HRD assay used, which may be the potential sources of bias. In future investigations, it might be of interest to evaluate the effectiveness and safety of PARP inhibitors according to HRD status in a maintenance setting (5). Secondly, a limited number of RCTs were included in the current work. Thirdly, variations in the follow‐up periods may have influenced the survival outcome measures. Fourthly, small phase 2 and large phase 3 trials were all included in the current study, which may be the potential sources of bias. Previous study suggests that when therapeutic interventions are associated with a potential for not common but serious adverse safety outcomes, there may be differences between small phase 2 and large phase 3 trials that result in their disagreement for safety but not necessarily efficacy outcomes (22).

Despite these limitations, our study has two critical strengths. Foremost, this is the first network meta-analysis comparing the efficacy and safety of single PARP inhibitors or combined with chemotherapy in a maintenance setting in platinum sensitive ROC. Secondly, we only included studies investigating patients with platinum sensitive ROC, and the baseline characteristics were similar among all the trials. Additionally, this meta-analysis stratified results according to BRCA mutational status: BRCAm population, HRD patients and the whole population. Stratified results allow us to better evaluate the extent of the efficacy of PARP inhibitors throughout different HRD phenotypes.

In conclusion, all the included maintenance treatment regimens are effective regardless of BRCA mutational status. No treatment was clearly superior to others between rucaparib, niraparib and olaparib in terms of PFS. The treatment discontinuations were not significantly different between the three drugs, although the fewest grade 3 or 4 adverse events were associated with olaparib. Clinicians should consider potential adverse events related to each of these interventions in clinical practice, and the adverse events are generally manageable.

## Data Availability Statement

The original contributions presented in the study are included in the article/**Supplementary Material**. Further inquiries can be directed to the corresponding authors.

## Author Contributions

Substantial contribution to the conception and design of the work: YX, LD, and LW. Analysis and interpretation of the data: LD, YT, and MB. Drafting the manuscript: YX and MB. Revising the work critically for important intellectual content: NH and LW. All authors contributed to the article and approved the submitted version.

## Conflict of Interest

The authors declare that the research was conducted in the absence of any commercial or financial relationships that could be construed as a potential conflict of interest.

## Supplementary Material

Supplementary Table 1 Risk of bias of included studies.
