# Supplementary material for: Comparative Efficacy and Safety of PARP Inhibitors as Maintenance Therapy in Platinum Sensitive Recurrent Ovarian Cancer: A Network Meta-Analysis
Source: Front Oncol. 2021 Feb 22;10:573801. doi: 10.3389/fonc.2020.573801 (PMC7937863; doi:10.3389/fonc.2020.573801)
Supplement: Supplementary Table 1 — Risk of bias of included studies. [file DataSheet_1.docx]

Supplement Table 1. Risk of bias of included studies.

| Author | Year | Adequate Sequence Generation (Selection Bias) | Allocation Concealment (Selection Bias) | Blinding of Participants and Researchers (Performance Bias) | Blinding of Outcome Assessment  (Detection Bias) | Incomplete Outcome Data (Attrition Bias) | Selective Reporting (Reporting Bias) | Other Bias |
| --- | --- | --- | --- | --- | --- | --- | --- | --- |
| Ledermann JA, et al.  (STUDY 19) | 2019 | + | + | + | + | + | ? | ? |
| Coleman RL, et al.  (VELIA/GOG-3005) | 2019 | + | + | + | + | + | + | ? |
| Pujade-Lauraine E, et al.  (SOLO2) | 2017 | + | + | + | + | + | + | ? |
| Coleman RL, et al.  (ARIEL3) | 2017 | + | + | + | + | + | + | ? |
| Mirza MR, et al.  (ENGOT-OV16/NOVA) | 2016 | ? | ? | + | + | + | - | ? |
| Oza AM, et al. | 2015 | ? | + | + | + | + | ? | ? |
